# Novel Waist-to-Height Ratio Estimated Fat Mass Pediatric Cut-offs Predict Hypertension Better than Body Mass Index in Multiracial United States Youths and Adults: The National Health and Nutrition Examination Survey 2015–2023 Cycle

**DOI:** 10.1016/j.tjnut.2026.101426

**Published:** 2026-02-18

**Authors:** Mahidere W Ali, Douglas R Corsi, Andrew O Agbaje

**Affiliations:** 1 Institute of Public Health and Clinical Nutrition, Faculty of Health Sciences, School of Medicine, University of Eastern Finland, Kuopio, Finland; 2 Department of Medicine, Rutgers Robert Wood Johnson Medical School, New Brunswick, NJ, United States; 3 Department of Public Health and Sports Sciences, Children’s Health and Exercise Research Centre, Faculty of Health and Life Sciences, University of Exeter, Exeter, United Kingdom

**Keywords:** obesity, cardiovascular diseases, children, preventive cardiology, metabolism

## Abstract

**Background:**

Emerging frameworks and clinical consensus statements have strongly recommended that diagnosing obesity with body mass index [BMI (in kg/m^2^)] should be confirmed with surrogate markers, such as the waist circumference-to-height ratio (WHtR). Recent studies have reported that new pediatric WHtR adiposity cutpoints predicted the risk of type 2 diabetes, fatty liver disease, and bone fracture in adults more accurately than BMI, but its role in predicting hypertension remains unclear.

**Objectives:**

To examine the associations of WHtR cutpoints with elevated blood pressure (BP) and hypertension among multiracial United States participants.

**Methods:**

Altogether 19,124 participants were studied from the United States National Health and Nutrition Examination Survey (NHANES) 2015–2023 cycle. WHtR cutoffs were defined as normal fat (0.40 to <0.50), high fat (0.50 to <0.53), and excess fat (≥0.53). Elevated BP was defined as ≥120/70 mm Hg, and hypertension as ≥140/90 mm Hg.

**Results:**

In the 2021–2023 NHANES cycle, participants’ mean age was 44.8 ± 22.6 y, elevated BP and hypertension prevalence were 63.5% and 14.4%, respectively. WHtR-high fat was associated with elevated BP [odds ratio (OR): 1.49; 95% confidence interval (CI): 1.04, 2.12, *P* < 0.001] and hypertension (OR: 1.82; 95% CI: 1.49, 2.22, *P* < 0.001), and WHtR-excess fat predicted higher odds of elevated BP (OR: 1.91; 95% CI: 1.40, 2.49, *P* < 0.001) and hypertension (OR: 2.61; 95% CI: 2.21, 3.08, *P* < 0.001). BMI-overweight (OR: 1.11; 95% CI: 0.90, 1.36, *P* = 0.321) and BMI-obesity (OR: 1.25; 95% CI: 1.00, 1.56, *P* = 0.058) were not associated with hypertension. In youth <25 y (mean age 14.7 y), both WHtR-high fat (OR: 1.70; 95% CI: 1.11, 2.61, *P* = 0.015) and WHtR-excess fat (OR: 2.16; 95% CI: 1.59, 2.92, *P* < 0.001) were associated with elevated BP, but not hypertension. WHtR-excess fat predicted elevated BP in non-Hispanic White, Blacks, and Asians. The results were consistent with 2015–2016, 2017–2018, and combined 2015–2023 NHANES cycles.

**Conclusions:**

The new pediatric WHtR-estimated adiposity cutpoints predicted the risk of elevated BP and hypertension across the lifecourse in a multiracial adult and youth population. WHtR is a universally accessible cardiovascular disease risk assessment tool for early screening, detection, prevention, and management of obesity and can be assessed at https://urfit-child.com/waist-height-calculator/.

## Introduction

The United States health system is presently burdened by both uncontrolled hypertension and unmanaged obesity [1,2]. According to the 2017 NHANES report, ∼49.5% of individuals with hypertension were classified as having obesity, whereas 35.7% of adults with obesity were noted to be hypertensive [1]. Although increased blood pressure (BP) is associated with a graded increase in cardiovascular disease (CVD) risk, the presence of multiple CVD risk factors in an individual significantly raises the absolute risk of future CVD events, both in children and adults [1,3,4]. A Framingham Offspring Study had previously determined adiposity as the single controllable precursor for high BP in young adults [5].

In children and adolescents, BP has a consistent and linear cross-sectional relationship with BMI levels; however, longitudinal evidence suggests that this correlation may not accurately represent the relationship of BP with adiposity due to the dynamics of body composition in children, where BMI largely reflects muscle mass [[6], [7], [8], [9]]. Clinical practice guidelines on the screening and management of obesity are largely based on the traditional BMI-guided classification, which is significantly limited by BMI variation across age, sex, ethnicity, and does not account for muscle mass [[10], [11], [12]]. Recently, the European Association for the Study of Obesity and the Lancet Commission recommended that the diagnoses of overweight and obesity must be confirmed with the waist circumference-to-height ratio (WHtR) ≥0.5, suggested as specific for excess adiposity [11,13,14]. However, the degree of adiposity accumulation, which may indicate risk severity, was unspecified in the scientific statements, necessitating the need for optimal cutoffs that will truly identify excess total and central fat mass [13,14].

Evidence from >400,000 UK Biobank adults suggested that guideline-recommended BP thresholds may overestimate elevated BP risks in the Black population and underestimate BP risks among South Asians [15]. A newly developed pediatric WHtR-adiposity cutoff has recently been validated as a better predictor of type 2 diabetes, bone fracture, and fatty liver diseases than BMI in adults, subsequently leading to the development of a WHtR calculator (https://urfit-child.com/waist-height-calculator/) [11,16‒18]. Moreover, WHtR estimated fat mass categories did not differ by race or ethnicity [17]. Therefore, the current study aims to externally validate the new WHtR-adiposity cutoff as a predictor of hypertension across several ethnicities and age groups and compare the ability of BMI and WHtR in predicting hypertension using data from the United States NHANES 2021‒2023, 2017–2018, and 2015–2016 cycles and combine the sample size of 2015–2023 NHANES cycles.

## Methods

### Study cohort

This study is based on the United States NHANES 2015–2016, 2017–2018, and 2021–2023 cycle data. This nationally representative study, conducted by the National Center for Health Statistics of the Centers for Disease Control and Prevention, assesses the health and nutritional status of adults and children in the United States. The study design and methods have been described in detail (https://www.cdc.gov/nchs/nhanes/about/index.html). The research ethics review board of the National Center for Health Statistics approved this survey protocol, and written informed consent was obtained from all participants by the NHANES. From the NHANES 2021–2023 cycle, we included 7243 participants aged ≥12 y who had complete BP and WHtR assessments. Of these, 8.5% are Mexican American, 55.2% are non-Hispanic White, 12.3% are non-Hispanic Black, 5.8% are non-Hispanic Asian, whereas other Hispanics and racial groups accounted for 18%. Additionally, a separate analysis was conducted among 1886 children, adolescents, and young adults under the age of 25. The original data used for this study are found in the Centers for Disease Control and Prevention data repository [19]. The NHANES 2017–2018 cycle included 6004 participants aged ≥12 y who had complete BP and WHtR assessments, whereas the 2015–2016 cycle had 6304 participants aged ≥12 y, resulting in 19,124 participants combined from 2015–2023 NHANES cycles.

### Anthropometry measurement

The NHANES anthropometry examination was conducted in a mobile examination center by 2 health technologists. Weight was recorded using a digital weighing scale, embedded on the exam room floor, height was measured using a digital stadiometer with a calibration system used for adjustment, and waist circumference was measured using a tape meter, above the iliac crest, and rounded to the nearest 0.1cm. WHtR was computed as waist circumference in centimetres divided by height in centimetres. In a previous study, the new WHtR cutoffs were validated among NHANES participants, and adiposity levels are defined as: normal fat (0.40 to <0.50 males, 0.40 to <0.51 females), high fat (0.50 to <0.53 males, 0.51 to <0.54 females), and excess fat (≥0.53 males, ≥0.54 females) [16]. BMI was calculated as weight in kilograms divided by height in centimetres squared.

### Cardiometabolic assessment

BP and heart rate were recorded for each eligible participant, measured from the right arm while seated. Three consecutive readings were taken after a 5-min rest period, each 1 min apart, using the Omron IntelliSense Blood Pressure Monitor (model: HEM-907XL). Cuff sizes were determined based on arm circumference. Systolic BP (SBP) and diastolic BP (DBP) were recorded as continuous variables. For this study, elevated BP was defined as SBP/DBP ≥120/70 mm Hg and hypertension ≥140/90 mm Hg [20]. We employed the same BP cutpoints in youth, in line with the pediatric clinical guideline [21]. Serum blood samples for total cholesterol and high-sensitivity C-reactive protein (hs-CRP) were collected and analysed per protocol with Roche/Hitachi Cobas 8000 analyser series, a fully automated, high-throughput laboratory system for clinical chemistry and immunology tests.

### Sociodemographic characteristics

Data on age, sex, educational status, smoking status, race, sedentary time, and physical activity were determined through a family and sample person demographics survey by trained interviewers using the computer-assisted personal interview system in the participant’s home or by telephone, using either English or Spanish language. Instruments used for this survey are publicly available [19].

### Statistical analysis

Participant characteristics were summarized using means with SDs for continuous variables and frequencies with percentages for categorical variables. Differences across WHtR categories were assessed using analysis of variance for continuous variables and χ^2^tests for categorical variables. The association between WHtR cutoff points and BP was assessed in 2 different approaches. First, linear regression was used to analyse the association between WHtR categories and SBP as a continuous variable. The normal fat mass category (WHtR 0.40 to <0.50) served as the reference group. Models were constructed hierarchically: model 1 was unadjusted; model 2 was model 1 adjusted for demographic factors (age, sex, race/ethnicity, and educational status); and model 3 was fully adjusted for demographic factors (model 2) in addition to clinical covariates (smoking status, physical activity, total cholesterol, and hs-CRP based on previous literature) [6,22,23].

Given the physiological differences in BP regulation across age groups, we conducted pre-specified age-stratified analyses examining WHtR associations within the following groups: 12‒24 y, 25‒45 y, 46‒65 y, and >65 y. These age categories were selected based on developmental and cardiovascular disease risk considerations. To assess the relative predictive performance of WHtR compared with BMI, we conducted parallel analyses using BMI categories as predictors of the same BP outcomes, with identical covariate adjustment strategies. We also conducted an ethnic specific analysis. The analyses were conducted for NHANES 2015–2016, 2017–2018, and 2021–2023 survey cycles separately and combined. To address missing covariate data, we employed multiple imputation using regression-based methods with 20 imputation cycles and 10 iterations per cycle. Little’s missing completely at random test was performed to evaluate missingness patterns. The multiple imputation procedure generated 20 complete datasets, and results were pooled using Rubin’s rules. A 2-sided *P* value of < 0.05 was assumed statistically significant. All statistical analyses were conducted using SPSS Statistics software, version 29.0 (IBM Corp.).

## Results

### Participants’ characteristics in the NHANES 2021–2023 cycle

This study involved 7243 participants who had complete BP and WHtR measurements. The mean (SD) age of the participants was 44.8 (22.6) y, and the mean (SD) WHtR was 0.58 (0.1) (Table 1). Of the participants, 24.6% had normal fat (WHtR 0.40‒220 <0.50), 9.7% high fat (WHtR 0.50 to <0.53), and 65.6% excess fat (WHtR >0.53), respectively. Both mean (SD) SBP [normal fat: 110.5 (14.4) mmHg; high fat: 118.0 (17.5) mm Hg; and excess fat: 122.3 (18.3) mm Hg, *P* < 0.001] and DBP [normal fat: 65.7 (9.7); high fat: 71.7 (10.9) mm Hg; and excess fat: 74.8 (11.1) mm Hg, *P* < 0.001] increased progressively across WHtR categories (Table 1). Similarly, there was a statistically significant rise in total cholesterol and hs-CRP concentrations, with a respective 19% and 74% increment observed in the excess fat category compared with the normal fat category, whereas smoking prevalence reduced with increasing fat mass. All characteristics of the participants described across WHtR categories are presented in Table 1.

**TABLE 1:** Characteristics of the NHANES cohort 2021–2023 cycle by waist circumference-to-height ratio adiposity cutpoints

Characteristic	WHtR (0.40 to <0.50) normal fat (*n* = 1783)	WHtR (0.50 to <0.53) high fat (*n* = 707)	WHtR (>0.53) excess fat (*n* = 4753)	*P* value for group difference
Age (y)	26.8 ± 19.0	43.0 ± 21.2	51.8 ± 20.1	<0.001
Sex [female, *n* (%)]	776 (43.5)	490 (69.3)	2615 (55.0)	<0.001
Height (cm)	162.7 ± 15.1	166.1 ± 11.7	165.4 ± 10.7	<0.001
Weight (kg)	54.1 (15.3)	66.3 (13.2)	87.4 (21.8)	<0.001
BMI (kg/m^2^)	20.0 (2.9)	23.8 (2.3)	31.7 (6.5)	<0.001
Waist circumference (cm)	73.4 ± 8.9	85.6 ± 6.2	106.0 ± 14.7	<0.001
Waist circumference-to-height ratio	0.45 ± 0.03	0.52 ± 0.01	0.64 ± 0.08	<0.001
Heart rate (bpm)	74.6 ± 12.8	71.8 ± 12.3	72.7 ± 12.4	<0.001
Systolic BP (mm Hg)	110.5 ± 14.4	118.0 ± 17.5	122.3 ± 18.3	<0.001
Diastolic BP (mm Hg)	65.7 ± 9.7	71.7 ± 10.9	74.8 ± 11.1	<0.001
Total cholesterol (mg/dL)	170.8 ± 40.1	194.4 ± 47.2	209.2 ± 51.3	<0.001
hs-CRP (mg/L)	1.19 ± 2.83	1.89 ± 3.76	4.52 ± 7.41	<0.001
Smoking status (do you now smoke cigarettes? yes)	628 (35.2)	247 (34.9)	1551 (32.6)	<0.001
Education, *n* (%)	—	—	—	<0.001
<9th grade	15 (1.8)	20 (3.6)	228 (5.3)	
9‒11th grade	49 (6.0)	29 (5.2)	355 (8.3)	
High school/GED	144 (17.6)	93 (16.6)	949 (22.2)	
Some college	207 (25.4)	153 (27.4)	1360 (31.9)	
College graduate or above	401 (49.1)	264 (47.2)	1373 (32.2)	
Race/ethnicity (%)	—	—	—	<0.001
Mexican American	141 (1.9)	65 (0.9)	413 (5.7)	—
Other Hispanic	201 (2.8)	60 (0.8)	545 (7.5)	—
Non-Hispanic White	908 (12.5)	393 (5.4)	2,700 (37.3)	—
Non-Hispanic Black	239 (3.3)	72 (1.0)	583 (8.0)	—
Non-Hispanic Asian	150 (2.1)	72 (1.0)	196 (2.7)	—
Other/multiracial	144 (2.0)	45 (0.6)	316 (4.4)	—
Sedentary time (min/d)	375 (204)	429 (937)	425 (790)	<0.001

Values are presented as mean (SD) for continuous variables and *n* (percentage) for categorical variables. *P* values for group differences were calculated using 1-way analysis of variance for continuous variables and Pearson’s χ^2^ test for categorical variables.

Abbreviations: BP, blood pressure; hs-CRP, high-sensitivity C-reactive protein; min, minimum; WHtR, waist circumference-to-height ratio; GED, general education development.

**FIGURE 1:**
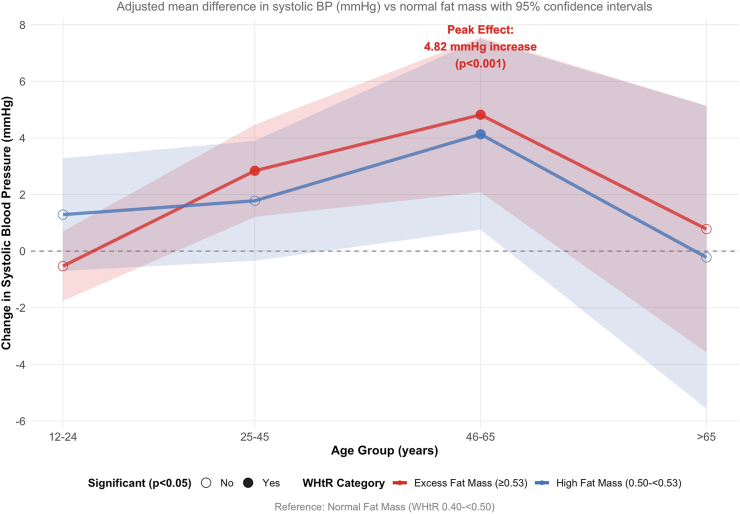
Age-stratified effects of waist circumference-to-height ratio (WHtR) on systolic blood pressure (BP). Age-stratified analysis of the differential effects of WHtR adiposity categories on systolic BP across the lifespan in 7243 adults from the NHANES 2021‒2023 cohort. The figure displays adjusted mean differences in systolic BP (mm Hg) with 95% confidence intervals (shaded areas) compared with the normal fat mass reference category (WHtR 0.40 to <0.50) across 4 age groups: 12‒24 y, 25‒45 y, 46‒65 y, and >65 y. Results are from linear regression models adjusted for age, sex, and race/ethnicity. The red line represents excess fat mass (WHtR ≥0.53) and the blue line represents high fat mass (WHtR 0.50 to <0.53). Filled circles indicate statistically significant associations (*P* < 0.05), whereas open circles represent non-significant findings. The analysis reveals a pronounced age-dependent pattern with peak cardiovascular impact occurring in middle-aged adults (46‒65 y), where excess fat mass was associated with a 4.82 mm Hg increase in systolic BP (*P* < 0.001). The effect diminishes in older adults (>65 y), suggesting age-related arterial changes may overshadow adiposity effects. No significant associations were observed in the youngest age group (12‒24 y) after adjustment, consistent with the predominant influence of lean mass on BP during growth and development. The horizontal dashed line at 0 represents no difference from the reference category.

The mean (SD) age of the 1886 participants <25 y was 14.7 (4.5) y. The mean (SD) WHtR was 0.51 (0.1). Among these youth participants, 58.1%, 9.8%, and 32.2% were categorised as having normal fat, high fat, and excess fat, respectively. The highest mean SBP was recorded among the WHtR high-fat category [107.5 (11.4), *P* = 0.019], and DBP among the excess fat category [67.4 (9.0), *P* < 0.001] (Figure 1 and Supplemental Tables 1 and 2). Mexican Americans, Non-Hispanic White and Non-Hispanic Black had a 3-fold higher prevalence of WHtR excess fat relative to normal fat (Table 1).

### Linear association of WHtR categories with SBP in NHANES 2021–2023 cycle

In the unadjusted model, WHtR categories were related to a higher SBP as a continuous outcome, with a SBP [7.54 mm Hg; 95% confidence interval (CI): 6.17, 8.91 mm Hg] in the WHtR high fat mass group and (11.78 mm Hg; 95% CI: 10.84, 12.72 mm Hg) in the excess fat mass group, which was markedly attenuated after adjusting for age and sex. In the fully adjusted model, excess fat mass was associated with higher SBP (1.30 mm Hg; 95% CI: 0.21, 2.39 mm Hg, *P* = 0.02) (Figure 1 and Table 2). In the age-stratified analysis adjusted for age and sex, excess fat increased SBP by 2.76 mm Hg among 25–45-y-olds, and by 4.82 mm Hg in those aged 45‒65 y. However, no significant increase was observed among individuals aged ≥65 y (Table 3). In participants age <25 y, the unadjusted analyses showed that high fat and excess fat WHtR categories were significantly associated with higher SBP compared with normal fat (high fat: 1.70 mm Hg; 95% CI: 0.65, 2.75 mm Hg, *P* = 0.043); and (excess fat: 1.27 mm Hg; 95% CI: 0.22, 2.32 mm Hg, *P* = 0.02), but was attenuated to statistical non-significance after full adjustment (Table 3).

**TABLE 2:** Associations of waist circumference-to-height ratio estimated fat mass cutpoints with continuous systolic blood pressure (NHANES 2021–2023 cycle)

*n* = 7243	Model 1		Model 2		Model 3	
WHtR category	β (95% CI)	*P* value	β (95% CI)	*P* value	β (95% CI)	*P* value
Normal fat mass	Reference		Reference		Reference	
High fat mass	7.54 (6.17, 8.91)	<0.001	1.01 (‒0.20, 2.22)	0.233	1.04 (‒0.22, 2.30)	0.688
Excess fat mass	11.78 (10.84, 12.72)	<0.001	1.75 (0.82, 2.68)	<0.001	1.30 (0.21, 2.39)	0.02

Model 1 was unadjusted, whereas model 2 was model 1 adjusted for age and sex. Model 3 was model 2 additionally adjusted for heart rate, educational status, smoking status, race, sedentary time, moderate physical activity, fasting total cholesterol, and high-sensitivity-C-reactive protein. A 2-sided *P* value < 0.05 was considered statistically significant.

Abbreviations: CI, confidence interval; WHtR, waist circumference-to-height ratio; β, unstandardized regression coefficient from linear regression models.

**TABLE 3:** Age-stratified associations of waist circumference-to-height ratio estimated fat mass cutpoints with continuous systolic blood pressure (NHANES 2021–2023 cycle)

Age group	WHtR fat mass category	Model 1		Model 2	
		β (95% CI)	*P* value	β (95% CI)	*P* value
12‒24.9 y					
	Normal	Reference	—	Reference	—
	High fat mass	1.29 (‒0.70, 3.28)	0.204	2.24 (0.41, 4.07)	0.063
	Excess fat mass	‒0.53 (‒1.76, 0.70)	0.4	0.55 (‒0.56, 1.66)	0.334
25‒45.9 y					
	Normal	Reference	—	Reference	—
	High fat mass	1.78 (–0.34, 3.90)	0.102	1.31 (–0.65, 3.27)	0.19
	Excess fat mass	2.84 (1.21, 4.47)	<0.001	2.76 (1.25, 4.27)	<0.001
46‒65 y					
	Normal	Reference	—	Reference	—
	High fat mass	4.13 (0.76, 7.50)	0.017	3.80 (0.47, 7.13)	0.026
	Excess fat mass	5.22 (2.48, 7.96)	<0.001	4.82 (2.10, 7.54)	<0.001
>65 y					
	Normal	Reference	—	Reference	—
	High fat mass	‒0.22 (–5.55, 5.11)	0.936	0.28 (–5.01, 5.57)	0.917
	Excess fat mass	0.78 (–3.55, 5.11)	0.724	0.60 (–3.71, 4.91)	0.784

Model 1: unadjusted linear regression. Model 2: adjusted for age, sex, and race/ethnicity. Comparisons are made within each age group, using the “Normal” WHtR category as the reference. *P* values are based on the regression model coefficients for each WHtR category compared with the reference. Associations are shown for categories of WHtR [normal fat (0.40 to <0.50), high fat (0.50 to <0.53), excess fat (≥0.53)], with “Normal fat” as the reference group. A 2-sided *P* value <0.05 was considered statistically significant. Estimates and 95% CIs are derived from linear regression models.

Abbreviations: CI, confidence interval; WHtR, waist circumference-to-height ratio.

### Association of WHtR cutpoints with elevated BP and hypertension in NHANES 2021–2023 cycle

**FIGURE 2:**
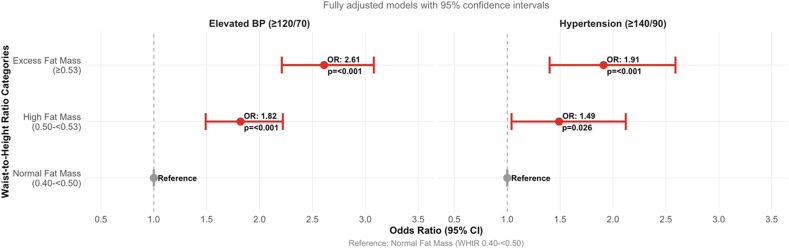
Forest plot of the relationship of blood pressure (BP) outcomes by waist circumference-to-height ratio (WHtR) categories. Forest plot of the association between WHtR adiposity categories and BP outcomes in 7243 adults from the NHANES 2021‒2023 cohort. The plot displays odds ratios (OR) with 95% confidence intervals (CI) for elevated BP (≥120/70 mm Hg) and hypertension (≥140/90 mm Hg) across WHtR categories. Results are from fully adjusted logistic regression models controlling for age, sex, heart rate, educational status, smoking status, race/ethnicity, sedentary time, moderate physical activity, fasting total cholesterol, and high-sensitivity C-reactive protein. The normal fat mass category (WHtR 0.40 to <0.50) serves as the reference group (OR = 1.0, indicated by gray circles). Red circles represent point estimates with horizontal lines indicating 95% CIs. The vertical dashed line at OR = 1.0 represents no association. For elevated BP, high fat mass (WHtR 0.50 to <0.53) was associated with (OR: 1.82; 95% CI: 1.49, 2.22, *P* < 0.001), and excess fat mass (WHtR ≥ 0.53) with (OR: 2.61; 95% CI: 2.21, 3.08, *P* < 0.001). For hypertension, high fat mass demonstrated (OR: 1.49; 95% CI: 1.04, 2.12, *P* = 0.026), and excess fat mass showed (OR: 1.91; 95% CI: 1.40, 2.59, *P* < 0.001). Statistical significance was defined as *P* < 0.05. The dose-response relationship demonstrates progressively increasing cardiovascular disease risk with higher WHtR adiposity categories.

Among the total participants, the prevalence of elevated BP was 63.5%, and 14.4% were hypertensive. In a logistic regression model fully adjusted for lifestyle and cardiometabolic factors, WHtR high fat increased odds of elevated BP by 49% [odds ratio (OR): 1.49; 95% CI: 1.04, 2.12, *P* < 0.001] and hypertension by 82% (OR: 1.82; 95% CI: 1.49, 2.22), *P* < 0.001), whereas excess fat was associated with 91% and 161% higher odd of elevated BP (OR: 1.91; 95% CI: 1.40, 2.59, *P* < 0.001) and hypertension (OR: 2.61; 95% CI: 2.21, 3.08, *P* < 0.001), respectively (Figure 2 and Table 4). Total cholesterol and hs-CRP were significant predictors of elevated BP but not hypertension after full adjustment, as shown in Table 4.

**TABLE 4:** Associations of waist circumference-to-height ratio cutpoints with elevated blood pressure and hypertension (NHANES 2021–2023 cycle)

*n* = 7243	Hypertension (≥140/90 mm Hg)		Elevated BP (≥120/70 mm Hg)	
Frequency (*n*, %)	1046/7243 (14.4)	—	4597/7243 (63.5)	—
Models	Odds ratio (95% CI)	*P* value	Odds ratio (95% CI)	*P* value
Model 1				
WHtR (0.40 to <0.50) normal fat	Reference	—	Reference	—
WHtR (0.50 to <0.53) high fat	2.86 (2.24, 3.66)	<0.001	2.90 (2.46, 3.42)	<0.001
WHtR (≥0.53) excess fat	5.35 (4.19, 6.83)	<0.001	5.58 (4.97, 6.27)	<0.001
Model 2				
WHtR (0.40 to <0.50) normal fat	Reference	—	Reference	—
WHtR (0.50 to <0.53) high fat	1.82 (1.49, 2.22)	<0.001	1.49 (1.04, 2.12)	0.001
WHtR (≥0.53) excess fat	2.61 (2.21, 3.08)	<0.001	1.91 (1.40, 2.59)	<0.001

Odds ratios and 95% CIs were estimated using logistic regression models. Model 1: unadjusted logistic regression. Model 2: logistic regression adjusted for age, sex, heart rate, educational status, smoking status, race/ethnicity, sedentary time, moderate physical activity, fasting total cholesterol, and high-sensitivity C-reactive protein. A 2-sided *P* value <0.05 was considered statistically significant.

Abbreviations: BP, blood pressure; CI, confidence interval; WHtR, waist circumference-to-height ratio.

In children and young adults age <25 y, elevated BP was present in 27% of participants, whereas only a few (1%) had hypertension. Both WHtR high fat (OR: 1.66; 95% CI: 1.14, 2.42, *P* = 0.009) and excess fat (OR: 1.98; 95% CI: 1.48, 2.65, *P* < 0.001) were strongly associated with elevated BP. The association detected between WHtR categories and hypertension was not significant after full adjustment (Figure 2 and Supplemental Table 3).

### Comparative analysis of WHtR compared with BMI in predicting elevated BP and hypertension in NHANES 2021–2023 cycle

**FIGURE 3:**
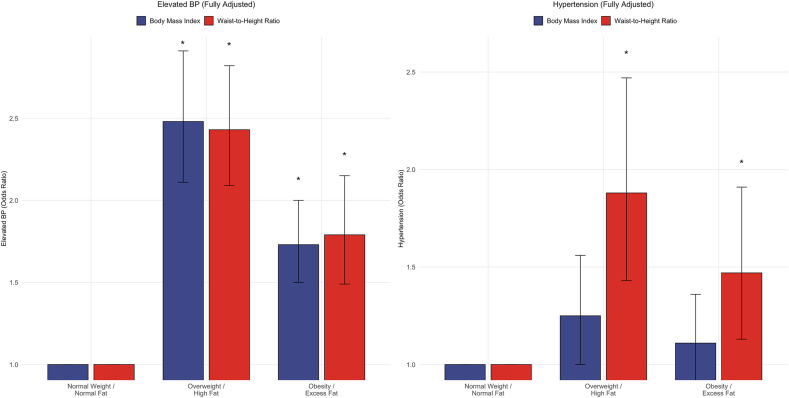
Comparative predictive performance of waist circumference-to-height ratio (WHtR) compared with BMI for blood pressure (BP) outcomes. Comparative analysis of the differential predictive performance of WHtR compared with BMI for BP outcomes in 7243 adults from the NHANES 2021‒2023 cohort. The figure displays odds ratios (OR) with 95% confidence intervals (CIs) (error bars) from fully adjusted logistic regression models for elevated BP (≥120/70 mm Hg, left panel) and hypertension (≥140/90 mm Hg, right panel). Dark blue bars represent BMI categories (normal weight, overweight, and obesity), whereas red bars represent WHtR categories (normal fat, high fat, and excess fat). All models were adjusted for age, sex, heart rate, educational status, smoking status, race/ethnicity, sedentary time, moderate physical activity, fasting total cholesterol, and high-sensitivity C-reactive protein. Asterisks (∗) indicate statistical significance (*P* < 0.05). For elevated BP, both WHtR and BMI demonstrated comparable predictive performance, with WHtR excess fat (OR: 1.91; 95% CI: 1.40, 2.59) and BMI obesity (OR: 2.48; 95% CI: 2.11, 2.91) showing strong associations. However, for hypertension prediction, WHtR demonstrated superior discriminatory ability with statistically significant associations across both high fat (OR: 1.82; 95% CI: 1.49, 2.22) and excess fat categories (OR: 2.61; 95% CI: 2.21, 3.08), whereas BMI categories failed to achieve statistical significance for hypertension prediction (overweight OR 1.11, *P* = 0.321; obesity OR 1.25, *P* = 0.058). This superior performance of WHtR for hypertension prediction supports its clinical utility as a more precise adiposity measure for cardiovascular disease risk stratification, particularly for established hypertensive disease compared with pre-hypertensive states.

In fully adjusted models, BMI overweight was associated with 71% higher odds (OR: 1.71; 95% CI: 1.48, 1.98, *P* < 0.001) and obesity with 130% higher odds (OR: 2.30; 95% CI: 1.90, 2.80, *P* < 0.001) of elevated BP (Supplemental Table 4). For hypertension, BMI categories showed weaker associations: overweight (OR: 1.11; 95% CI: 0.90, 1.36, *P* = 0.321) and obesity (OR: 1.25; 95% CI: 1.00, 1.56, *P* = 0.0.058). WHtR demonstrated superior discriminatory ability compared with BMI (Supplemental Table 4). The excess fat mass WHtR category showed stronger associations with both elevated BP and hypertension compared with BMI obesity categories, particularly for hypertension prediction, where BMI categories failed to reach statistical significance (Figure 3 and Supplemental Table 4).

### Racial/ethnicity-based association of WHtR cutpoints with elevated BP and hypertension in NHANES 2021–2023 cycle

In fully adjusted models, excess fat mass WHtR category was associated with higher odds of elevated BP among non-Hispanic White (OR: 2.85; 95% CI: 2.25, 3.61, *P* < 0.001), non-Hispanic Black (OR: 2.73; 95% CI: 1.80, 4.15, *P* < 0.001), non-Hispanic Asians (OR: 4.27; 95% CI: 2.26, 8.08, *P* < 0.001), other Hispanics (OR: 1.75; 95% CI: 1.14, 2.69, *P* = 0.01) and other/multiracial groups (OR: 2.71; 95% CI: 1.56, 4.71, *P* < 0.001) but not between Mexican Americans (OR: 1.70; 95% CI: 0.91, 3.18) (Table 5). The high fat mass WHtR category was associated with higher odds of elevated BP among non-Hispanic White, non-Hispanic Asians, and other/multiracial groups, but not between Mexican American and non-Hispanic Black (Table 5). Excess fat mass WHtR category was associated with higher odds of hypertension only among non-Hispanic White but not between other racial groups. High fat mass WHtR category was not associated with hypertension across all ethnic/racial groups (Table 5).

**TABLE 5:** Associations of waist circumference-to-height ratio cutoffs with elevated blood pressure and hypertension by race and Hispanic origin (NHANES 2021–2023 cycle)

Race/ethnicity	Hypertension (≥140/90 mm Hg)				Elevated BP (≥120/70 mm Hg)			
	Odds ratio (95% CI)	*P* value	Odds ratio (95% CI)	*P* value	Odds ratio (95% CI)	*P* value	Odds ratio (95% CI)	*P* value
	Model 1		Model 2		Model 1		Model 2	
Mexican American								
WHtR normal fat	Reference	—	Reference	—	Reference	—	Reference	—
WHtR high fat	3.36 (0.55, 20.69)	0.19	1.31 (0.17, 9.91)	0.8	2.06 (1.07, 4)	0.032	0.74 (0.32, 1.70)	0.47
WHtR excess fat	10.45 (2.51, 43.55)	0.0013	2.20 (0.41, 11.83)	0.36	6.72 (4.24, 10.64)	<0.001	1.70 (0.91, 3.18)	0.094
Other Hispanic								
WHtR normal fat	Reference	—	Reference	—	Reference	—	Reference	—
WHtR high fat	0.83 (0.09, 7.64)	0.873	0.38 (0.03, 4.37)	0.43	2.1 (1.16, 3.78)	0.014	1.23 (0.63, 2.40)	0.54
WHtR excess fat	9.36 (3.38, 25.88)	<0.001	2.63 (0.68, 10.17)	0.16	4.36 (3.08, 6.18)	<0.001	1.75 (1.14, 2.69)	0.01
Non-Hispanic White								
WHtR normal fat	Reference	—	Reference	—	Reference	—	Reference	—
WHtR high fat	2.87 (1.86, 4.45)	<0.001	1.58 (0.93, 2.69)	0.09	3.04 (2.37, 3.89)	<0.001	2.00 (1.51, 2.65)	<0.001
WHtR excess fat	4.83 (3.48, 6.7)	<0.001	1.90 (1.13, 3.17)	0.016	5.68 (4.84, 6.68)	<0.001	2.85 (2.25, 3.61)	<0.001
Non-Hispanic Black								
WHtR normal fat	Reference	—	Reference	—	Reference	—	Reference	—
WHtR high fat	3.67 (1.71, 7.87)	<0.001	1.65 (0.65, 4.21)	0.3	2.83 (1.63, 4.89)	<0.001	1.42 (0.75, 2.70)	0.28
WHtR excess fat	4.74 (2.76, 8.15)	<0.001	1.94 (0.94, 4.04)	0.075	5.91 (4.26, 8.21)	<0.001	2.73 (1.80, 4.15)	<0.001
Non-Hispanic Asian								
WHtR normal fat	Reference	—	Reference	—	Reference	—	Reference	—
WHtR high fat	4.56 (1.32, 15.76)	0.017	2.35 (0.44, 12.62)	0.32	4.82 (2.63, 8.83)	<0.001	2.71 (1.32, 5.54)	0.007
WHtR excess fat	9.36 (3.26, 26.89)	<0.001	2.92 (0.71, 11.93)	0.13	9.72 (5.89, 16.02)	<0.001	4.27 (2.26, 8.08)	<0.001
Other/multiracial								
WHtR normal fat	Reference	—	Reference	—	Reference	—	Reference	—
WHtR high fat	1.91 (0.53, 6.87)	0.321	1.24 (0.29, 5.25)	0.77	3.62 (1.79, 7.33)	<0.001	2.69 (1.21, 5.97)	0.015
WHtR excess fat	4.87 (2.17, 10.96)	<0.001	2.28 (0.82, 6.35)	0.11	5.02 (3.28, 7.68)	<0.001	2.71 (1.56, 4.71)	<0.001

Odds ratios and 95% CIs were estimated using logistic regression models. Model 1: unadjusted logistic regression. Model 2: logistic regression adjusted for age, sex, heart rate, educational status, smoking status, sedentary time, moderate physical activity, fasting total cholesterol, and high-sensitivity C-reactive protein. A 2-sided *P* value <0.05 was considered statistically significant and is bolded. Associations are shown for categories of WHtR (normal fat (0.40 to <0.50), high fat (0.50 to <0.53), excess fat (≥0.53), with “Normal fat” as the reference group.

Abbreviations: BP, blood pressure; CI, confidence interval; WHtR, waist circumference-to-height ratio.

### NHANES 2017–2018 and the combined 2017–2023 NHANES characteristics

Altogether, 69% of 12,820 participants combined from NHANES 2017–2018 and 2021–2023 cycles had WHtR excess fat mass, were significantly older with a mean age of 51.8 y compared with 28.6 y among those with WHtR normal fat mass. Participants with excess fat mass had statistically significantly higher BP, prevalence of hypertension, higher mean BMI, higher cholesterol, glucose, and inflammatory marker concentrations (Table 6). The demographic characteristics were consistent with the separate NHANES 2017–2018 and 2021–2023 cycles (TABLE 1, TABLE 7). Among youths between ages 12 and <25 y, in the combined NHANES 2017–2018 and 2021–2023 cycles, 35.9% of the 2819 participants had WHtR excess fat mass compared with 50.3% with WHtR normal fat mass (Supplemental Table 5). The prevalence was consistent with the individual NHANES 2017–2018 cycle (Supplemental Table 6).

**TABLE 6:** Characteristics of combined NHANES 2017–2018 and 2021–2023 participants aged ≥12 y, by waist circumference-to-height ratio adiposity categories

Characteristic	Normal fat (*n* = 2455)	High fat mass (*n* = 1517)	Excess fat mass (*n* = 8848)	*P* value
Age, y, mean (SD)	28.59 (17.71)	42.08 (20.26)	51.76 (19.13)	<0.001
Systolic BP, mm Hg, mean (SD)	112.89 (14.76)	119.16 (17.38)	123.92 (18.81)	<0.001
Diastolic BP, mm Hg, mean (SD)	66.67 (9.65)	71.76 (10.28)	75.10 (11.25)	<0.001
Elevated blood pressure, *n* (%)	884 (36.0)	882 (58.1)	6342 (71.7)	<0.001
Hypertension, *n* (%)	126 (5.1)	177 (11.7)	1786 (20.2)	<0.001
Waist circumference, cm, mean (SD)	74.83 (6.57)	85.36 (5.54)	106.52 (14.40)	<0.001
Height, cm, mean (SD)	167.97 (10.10)	167.22 (10.07)	165.94 (10.03)	<0.001
BMI, kg/m^2^, mean (SD)	20.75 (2.50)	24.01 (2.18)	31.95 (6.58)	<0.001
Sex, *n* (%)				<0.001
Male	1294 (52.7)	712 (46.9)	4081 (46.1)	—
Female	1161 (47.3)	805 (53.1)	4767 (53.9)	—
Race/ethnicity, *n* (%)				<0.001
Mexican American	212 (8.6)	136 (9.0)	1069 (12.1)	—
Other Hispanic	215 (8.8)	131 (8.6)	936 (10.6)	—
Non-Hispanic White	1042 (42.4)	698 (46.0)	4121 (46.6)	—
Non-Hispanic Black	495 (20.2)	221 (14.6)	1525 (17.2)	—
Other race/multiracial	491 (20.0)	331 (21.8)	1197 (13.5)	—
Education, *n* (%)^1^^,^^2^				<0.001
<9th grade	27 (2.1)	45 (3.7)	595 (7.3)	—
9–11th grade	117 (9.1)	92 (7.6)	793 (9.7)	—
High school/GED	269 (20.9)	246 (20.2)	1870 (23.0)	—
Some college/AA degree	372 (28.9)	325 (26.7)	2639 (32.4)	—
College graduate or above	503 (39.1)	507 (41.7)	2240 (27.5)	—
Refused	0 (0.0)	1 (0.1)	1 (0.0)	—
Don’t know	0 (0.0)	0 (0.0)	9 (0.1)	—
Income-to-poverty ratio, mean (SD)	2.62 (1.69)	2.83 (1.72)	2.66 (1.62)	0.001
Total cholesterol, mg/dL, mean (SD)	166.62 (36.57)	183.39 (40.46)	187.70 (42.45)	<0.001
Glucose, mg/dL, mean (SD)	97.57 (22.56)	101.69 (26.10)	113.89 (36.31)	<0.001
hs-CRP, mg/L, mean (SD)	1.56 (5.89)	1.97 (4.11)	4.49 (8.02)	<0.001
Sedentary time, min/d, mean (SD)	388.86 (680.51)	376.98 (686.46)	414.25 (809.34)	0.178
Physical activity (harmonized), mean (SD)	75.36 (104.72)	104.78 (456.95)	128.46 (597.78)	0.002

Abbreviations: BP, blood pressure; hs-CRP, high-sensitivity C-reactive protein; min, minimum; GED; AA degree.

1 Education among participants aged ≥20 y; categories as defined in NHANES.

2 Percentages may not sum to 100 due to rounding.

**TABLE 7:** Characteristics of NHANES 2017–2018 participants aged ≥12 y, by waist circumference-to-height ratio adiposity categories

Characteristic	Normal fat (*n* = 1192)	High fat mass (*n* = 660)	Excess fat mass (*n* = 4152)	*P* value
Age, y, mean (SD)	27.64 (16.99)	39.88 (20.26)	50.23 (19.27)	<0.001
Systolic BP, mm Hg, mean (SD)	113.59 (15.38)	119.89 (17.33)	125.10 (19.57)	<0.001
Diastolic BP, mm Hg, mean (SD)	66.70 (9.93)	71.22 (9.79)	75.03 (11.54)	<0.001
Elevated BP, *n* (%)	405 (34.0)	358 (54.2)	2813 (67.8)	<0.001
Hypertension, *n* (%)	70 (5.9)	79 (12.0)	887 (21.4)	<0.001
Waist circumference, cm, mean (SD)	74.46 (6.48)	85.16 (5.55)	106.29 (14.32)	<0.001
Height, cm, mean (SD)	167.53 (10.05)	166.79 (10.01)	165.70 (10.10)	<0.001
BMI, kg/m^2^, mean (SD)	20.68 (2.47)	24.00 (2.21)	31.93 (6.52)	<0.001
Sex, *n* (%)				<0.001
Male	654 (54.9)	318 (48.2)	1983 (47.8)	—
Female	538 (45.1)	342 (51.8)	2169 (52.2)	—
Race/ethnicity, *n* (%)				<0.001
Mexican American	118 (9.9)	72 (10.9)	668 (16.1)	—
Other Hispanic	79 (6.6)	49 (7.4)	404 (9.7)	—
Non-Hispanic White	380 (31.9)	222 (33.6)	1435 (34.6)	—
Non-Hispanic Black	333 (27.9)	122 (18.5)	946 (22.8)	—
Other race/multiracial	282 (23.7)	195 (29.5)	699 (16.8)	—
Education, *n* (%)^1^^,^^2^				<0.001
<9th grade	16 (2.6)	22 (4.3)	361 (9.5)	—
9–11th grade	76 (12.3)	55 (10.8)	428 (11.3)	—
High school/GED	148 (23.9)	127 (25.0)	904 (23.8)	—
Some college/AA degree	199 (32.2)	133 (26.2)	1252 (33.0)	—
College graduate or above	179 (29.0)	169 (33.3)	840 (22.2)	—
Refused	0 (0.0)	1 (0.2)	1 (0.0)	—
Don’t know	0 (0.0)	0 (0.0)	6 (0.2)	—
Income-to-poverty ratio, mean (SD)	2.43 (1.63)	2.53 (1.66)	2.49 (1.58)	0.389
Total cholesterol, mg/dL, mean (SD)	165.86 (35.27)	179.91 (39.77)	187.96 (41.76)	<0.001
Glucose, mg/dL, mean (SD)	97.49 (12.93)	103.59 (22.05)	117.01 (40.11)	<0.001
hs-CRP, mg/L, mean (SD)	1.73 (5.90)	1.92 (4.54)	4.58 (8.23)	<0.001
Sedentary time, min/d, mean (SD)	373.90 (736.77)	346.58 (618.86)	390.71 (768.49)	0.407
Physical activity (harmonized), mean (SD)	83.02 (135.39)	117.29 (139.98)	190.38 (650.49)	<0.001

Abbreviations: BP, blood pressure; hs-CRP, high-sensitivity C-reactive protein; min, minimum; GED; AA degree.

1 Education among participants aged ≥20 y; categories as defined in NHANES.

2 Percentages may not sum to 100 due to rounding.

### Association of WHtR categories with hypertension and elevated BP in 2017–2018 NHANES cycle

The WHtR category was not independently associated with mean SBP after multivariable adjustment; compared with normal WHtR, high fat mass and excess fat mass were associated with small, nonsignificant differences in SBP (∼1.0 and 0.8 mm Hg higher, respectively; both *P* > 0.20). Older age was strongly associated with higher SBP (∼0.46 mm Hg/y, *P* < 0.001), and females had lower SBP than males by roughly 5.2 mm Hg (*P* < 0.001). Excess WHtR fat mass was strongly associated with elevated BP (OR: 2.62; 95% CI: 2.18, 3.15, *P* < 0.001), after multivariable adjustment. Also, high WHtR fat mass was associated with elevated BP (OR: 1.80; 95% CI: 1.42, 2.28, *P* < 0.001). Excess WHtR fat mass was associated with higher odds of hypertension (OR: 1.61; 95% CI: 1.19, 2.17, *P* = 0.001), after multivariable adjustment, whereas the high–fat-mass WHtR category showed no clear difference compared with normal WHtR (OR: 1.20; 95% CI: 0.83, 1.72, *P* = 0.34). Among 2017–2018 NHANES cycle youths ages 12 to <25 y old, WHtR excess fat mass was associated with elevated BP (OR: 2.13; 95% CI: 1.48, 3.07, *P* < 0.001), whereas WHtR high fat mass association with elevated BP tended towards significance (OR: 1.54; 95% CI: 0.98, 2.41, *P* = 0.064) after full adjustments. The associations of either WHtR excess or high fat mass with hypertension were not statistically significant among youths.

### Association of WHtR categories with hypertension and elevated BP in 2017–2023 NHANES cycle

In the pooled cohort across survey 2017–2023 cycles, adults with excess WHtR had mean SBP ∼1.1 mm Hg higher than those with normal WHtR fat mass (*P* = 0.01), whereas those with high WHtR fat mass had SBP ∼0.8 mm Hg higher, but not statistically significant (*P* = 0.16). Age remained strongly positively associated with SBP, females had substantially lower SBP than males, and higher educational attainment was associated with lower SBP, with the largest reduction among college graduates.

Excess WHtR fat mass was associated with higher odds of elevated BP (OR: 2.76; 95% CI: 2.45, 3.10, *P* < 0.001), after multivariable adjustment. WHtR high fat mass was also associated with higher odds of elevated BP (OR: 1.80; 95% CI: 1.55, 2.09, *P* < 0.001). Excess WHtR fat mass was associated with higher odds of hypertension (OR: 1.81; 95% CI: 1.47, 2.23, *P* < 0.001), after multivariable adjustment. Similarly, WHtR high fat mass was associated with higher odds of hypertension (OR: 1.36; 95% CI: 1.05, 1.77, *P* = 0.02). Among youths ages 12 to <25 y old in the combined NHANES cohort 2017–2023 cycle, WHtR excess fat mass was associated with elevated BP (OR: 2.22; 95% CI: 1.75, 2.81, *P* < 0.001), and WHtR high fat mass was associated with elevated BP (OR: 1.81; 95% CI: 1.37, 2.40, *P* < 0.001). Among youths ages 12 to <25 y old in the combined NHANES cohort 2017–2023 cycle, WHtR excess fat mass was not associated with hypertension (OR: 1.39; 95% CI: 0.57, 3.39, *P* = 0.46), and WHtR high fat mass was not associated with hypertension (OR: 1.48; 95% CI: 0.49, 4.47, *P* = 0.49).

### NHANES 2015–2016 and the combined 2015–2023 NHANES characteristics

Altogether, 69% of 19,124 participants combined from NHANES 2015–2016, 2017–2018, and 2021–2023 cycles had WHtR excess fat mass, were significantly older with a mean age of 50.7 y compared with 25.7 y among those with WHtR normal fat mass. Participants with excess fat mass had statistically significantly higher BP, prevalence of hypertension, higher mean BMI, higher cholesterol, glucose, and inflammatory marker concentrations (Table 8). The demographic characteristics were consistent with the separate NHANES 2015–2016, 2017–2018, and 2021–2023 cycles (TABLE 1, TABLE 7, TABLE 9).

**TABLE 8:** Characteristics of the combined NHANES 2015–2016, 2017–2018, and 2021–2023 participants aged ≥12 y, by waist circumference-to-height ratio adiposity categories

Characteristic	Normal fat (*n* = 3755)	High fat mass (*n* = 2249)	Excess fat mass (*n* = 13,120)	*P* value
Age, y, mean (SD)	27.52 (16.96)	40.89 (19.96)	50.69 (19.19)	<0.001
Systolic BP, mm Hg, mean (SD)	112.64 (14.25)	119.04 (17.15)	124.62 (18.55)	<0.001
Diastolic BP, mm Hg, mean (SD)	65.50 (10.43)	70.47 (10.93)	73.13 (12.24)	<0.001
Elevated BP, *n* (%)	1354 (38.1)	1297 (60.3)	9423 (75.0)	<0.001
Hypertension, *n* (%)	177 (5.0)	250 (11.6)	2639 (21.0)	<0.001
Waist circumference, cm, mean (SD)	74.74 (6.48)	85.26 (5.45)	106.20 (14.37)	<0.001
Height, cm, mean (SD)	167.77 (10.03)	167.07 (9.95)	165.72 (10.04)	<0.001
BMI, kg/m^2^, mean (SD)	20.75 (2.47)	23.99 (2.20)	31.85 (6.52)	<0.001
Sex, *n* (%)				
Male	2031 (54.1)	1076 (47.8)	6067 (46.2)	<0.001
Female	1724 (45.9)	1173 (52.2)	7053 (53.8)	—
Race/ethnicity, *n* (%)				
Mexican American	346 (9.2)	239 (10.6)	1979 (15.1)	<0.001
Other Hispanic	340 (9.1)	214 (9.5)	1567 (11.9)	—
Non-Hispanic White	1452 (38.7)	930 (41.4)	5485 (41.8)	—
Non-Hispanic Black	839 (22.3)	360 (16.0)	2387 (18.2)	—
Other race	778 (20.7)	506 (22.5)	1702 (13.0)	—
Education, *n* (%)^1,2^				<0.001
<9th grade	55 (2.9)	78 (4.3)	1122 (9.3)	—
9–11th grade	178 (9.3)	154 (8.6)	1264 (10.5)	—
High school/GED	380 (19.9)	351 (19.6)	2762 (22.9)	—
Some college/AA degree	563 (29.6)	505 (28.1)	3795 (31.5)	—
College graduate or above	729 (38.3)	705 (39.3)	3092 (25.7)	—
Family PIR, mean (SD)	2.54 (1.65)	2.74 (1.70)	2.55 (1.61)	<0.001
Total cholesterol, mg/dL, mean (SD)	165.59 (35.58)	183.14 (41.44)	188.49 (42.36)	<0.001
Glucose, mg/dL, mean (SD)	97.39 (22.12)	101.89 (26.62)	114.26 (37.89)	<0.001
hs-CRP, mg/L, mean (SD)	1.43 (5.19)	2.02 (4.73)	4.54 (7.88)	<0.001
Sedentary time mean (SD)	423.56 (801.59)	409.96 (767.78)	441.92 (771.51)	0.36
Physical activity mean (SD)	78.79 (115.17)	113.53 (407.03)	144.46 (621.48)	<0.001

Abbreviations: BP, blood pressure; hs-CRP, high-sensitivity C-reactive protein; AA; GED; PIR.

**TABLE 9:** Characteristics of NHANES 2015–2016 participants aged ≥12 y, by waist circumference-to-height ratio adiposity categories

Characteristic	Normal fat (*n* = 1300)	High fat mass (*n* = 732)	Excess fat mass (*n* = 4272)	*P* value
Age, y, mean (SD)	25.51 (15.25)	38.42 (19.12)	48.48 (19.12)	<0.001
Systolic BP, mm Hg, mean (SD)	112.18 (13.28)	118.81 (16.70)	126.00 (17.94)	<0.001
Diastolic BP, mm Hg, mean (SD)	63.38 (11.42)	67.84 (11.71)	69.23 (13.15)	<0.001
Elevated BP, *n* (%)	470 (36.2)	415 (56.7)	3081 (72.1)	<0.001
Hypertension, *n* (%)	51 (3.9)	73 (10.0)	853 (20.0)	<0.001
Waist circumference, cm, mean (SD)	74.59 (6.30)	85.04 (5.26)	105.53 (14.28)	<0.001
Height, cm, mean (SD)	167.41 (9.91)	166.77 (9.68)	165.27 (10.05)	<0.001
BMI, kg/m^2^, mean (SD)	20.74 (2.41)	23.93 (2.22)	31.64 (6.39)	<0.001
Sex *n* (%)				<0.001
Male	737 (56.7)	364 (49.7)	1986 (46.5)	—
Female	563 (43.3)	368 (50.3)	2286 (53.5)	—
Race/ethnicity, *n* (%)				
Mexican American	134 (10.3)	103 (14.1)	910 (21.3)	<0.001
Other Hispanic	125 (9.6)	83 (11.3)	631 (14.8)	—
Non-Hispanic White	410 (31.5)	232 (31.7)	1364 (31.9)	—
Non-Hispanic Black	344 (26.5)	139 (19.0)	862 (20.2)	—
Other race	287 (22.1)	175 (23.9)	505 (11.8)	—
Education, *n* (%)^1,2^				<0.001
<9th grade	28 (4.5)	33 (5.7)	527 (13.5)	—
9–11th grade	61 (9.9)	62 (10.7)	471 (12.1)	—
High school/GED	111 (18.0)	105 (18.2)	892 (22.9)	—
Some college degree	191 (31.0)	180 (31.1)	1156 (29.6)	—
College graduate or above	226 (36.6)	198 (34.3)	852 (21.9)	—
Family PIR, mean (SD)	2.40 (1.57)	2.56 (1.64)	2.33 (1.58)	0.002
Total cholesterol, mg/dL, mean (SD)	163.68 (33.60)	182.64 (43.39)	190.09 (42.13)	<0.001
Glucose, mg/dL, mean (SD)	97.03 (21.22)	102.40 (27.94)	115.09 (41.17)	<0.001
hs-CRP, mg/L, mean (SD)	1.18 (3.53)	2.13 (5.79)	4.65 (7.59)	<0.001
Sedentary time mean (SD)	441.75 (786.00)	468.46 (891.56)	505.17 (863.64)	0.047
Physical activity (pa_var), mean (SD)	86.27 (134.91)	142.07 (155.22)	194.85 (688.55)	<0.001

Abbreviations: BP, blood pressure; hs-CRP, high-sensitivity C-reactive protein; GED, general education development; PIR, ratio of family income to poverty.

### Association of WHtR categories with hypertension and elevated BP in 2015–2016 NHANES cycle

The WHtR category was not independently associated with mean SBP after multivariable adjustment; compared with WHtR normal fat mass, WHtR high fat mass and WHtR excess fat mass were associated with small, nonsignificant differences in SBP (∼1.0 and 0.8 mm Hg higher, respectively; both *P* > 0.20). Older age was strongly associated with higher SBP (∼0.46 mm Hg/y, *P* < 0.001), and females had lower SBP than males by roughly 5.2 mm Hg (*P* < 0.001). WHtR excess fat mass was strongly associated with elevated BP (OR: 2.62; 95% CI: 2.18, 3.15, *P* < 0.001), after multivariable adjustment. Also, WHtR high fat mass was associated with elevated BP (OR: 1.80; 95% CI: 1.42, 2.28, *P* < 0.001). WHtR excess fat mass was associated with higher odds of hypertension (OR: 1.61; 95% CI: 1.19, 2.17, *P* = 0.001), after multivariable adjustment, whereas the WHtR high fat mass showed no clear difference compared with WHtR normal fat mass (OR: 1.20; 95% CI: 0.83, 1.72, *P* = 0.34).

### Association of WHtR categories with hypertension and elevated BP in 2017–2023 NHANES cycle

In the pooled cohort across survey 2017–2023 cycles, adults with excess WHtR had mean SBP ∼1.1 mm Hg higher than those with normal WHtR fat mass (*P* = 0.01), whereas those with high WHtR fat mass had SBP ∼0.8 mm Hg higher, but not statistically significant (*P* = 0.16). Age remained strongly positively associated with SBP, females had substantially lower SBP than males, and higher educational attainment was associated with lower SBP, with the largest reduction among college graduates.

WHtR excess fat mass was associated with higher odds of elevated BP (OR: 2.48; 95% CI: 2.23, 2.76, *P* < 0.001), after multivariable adjustment. WHtR high fat mass was also associated with higher odds of elevated BP (OR: 1.7; 95% CI: 1.51, 1.92, *P* < 0.001). WHtR excess fat mass was associated with higher odds of hypertension (OR: 1.83; 95% CI: 1.53, 2.19, *P* < 0.001), after multivariable adjustment. Similarly, WHtR high fat mass was associated with higher odds of hypertension (OR: 1.33; 95% CI: 1.07, 1.65, *P* = 0.01).

## Discussion

In the present study, we examined the relationship of the new WHtR pediatric adiposity cutoff with the risk of elevated BP and hypertension in the United States multiracial population. This study externally validated the new WHtR estimated fat mass category in predicting SBP and DBP, markers of CVD risk [16]. We observed a graded BP increase across WHtR fat mass categories, and both high and excess fat WHtR categories were significantly associated with increased odds of elevated BP across several races and hypertension only among the non-Hispanic White. Our study identified age as an effect modifier, such that a stronger association was found in middle-aged adults, but not in the elderly, likely because BP among the elderly was lower. In youth (<25 y), WHtR was not significantly associated with higher SBP continuous measures after full model adjustment. However, WHtR high and excess fat groups still predicted increased odds of elevated BP in youth, but not hypertension in each NHANES 2017–2018 and 2021–2023 cycle and in the combined sample size of ∼2800 youths, likely due to the very low prevalence (1.4%) of hypertension in this age group.

A systematic review and meta-analysis conducted in 2018 examined the impact of various anthropometric measures of body fat on the risk of hypertension [24]. The WHtR segment of the analysis that included 18,910 adult participants found a 1.74 mm Hg (95% CI: 1.35, 2.13 mm Hg) rise in BP for every 0.1 unit increase in WHtR [24]. This systematic review concluded that WHtR had the strongest discriminatory power in detecting hypertension compared with BMI, waist circumference, and waist-to-hip ratio [24,25]. It is noteworthy that most studies were conducted among the Asian population; however, the positive association found in the present study is in line with previous studies [22,23,26,27]. For example, a dose-dependent linear association was identified in a subset of the China hypertension survey study. This study reported (OR: 1.25; 95% CI: 1.08, 1.44, *P* = 0.002) for developing hypertension in the second WHtR tertile (0.47, 0.52) and (OR: 2.48; 95% CI: 2.18, 2.83, *P* < 0.001) in the third WHtR tertile (≥0.52). Furthermore, the study found a weaker association among participants aged ≥60 compared with younger adults [22]. A recent large-scale study involving Finnish and United Kingdom adults with >440,000 participants concluded that neither BMI nor waist-to-hip ratio on its own exceeded the accuracy of WHtR, because WHtR was a better predictor of type 2 diabetes and heart disease than either of the 2 alone [28].

Essential or primary hypertension (90% of the cases) is known to progress as a function of age, and excess adiposity has been identified as the greatest modifiable risk factor, influenced by sedentariness and dietary behaviour [29,30]. Ageing is accompanied by a gradual vascular remodelling, endothelial dysfunction, and fibrosis; this progression markedly accelerates beyond the age of 60, especially in the large arteries, contributing to the isolated systolic hypertension of old age [[31], [32], [33]]. Adiposity (excess fat mass) and adiposopathy (dysfunctional adipose tissue turnover and proliferation) contribute to this vascular aging process through direct lipotoxicity, hyper-inflammatory state, dysregulated vasoactive substances secretion, and fat deposit pressure on vital organs [34]. In our study, the strongest influence of adiposity on SBP was observed in middle-aged adults. In old age, the effect of adiposity was attenuated, suggesting the establishment of age-related irreversible arterial stiffness [32,33]. Hence, blunting the progress of hypertension through management of excess adiposity at the earliest detectable point in life may abate or reverse ongoing cardiovascular disease risk [35].

In children and adolescents, future adverse CV outcomes are well documented among those with elevated BP [3]. The high prevalence of elevated BP (27%) and excess fat (32.2%) found in this United States youth population with a mean age of 14 y is of clinical significance when compared with a prevalence of elevated SBP of 11.6% in the 17-y-old United Kingdom population [4]. It is well known that elevated BP in childhood often progresses to hypertension in adulthood, and obesity similarly persists into adulthood in the majority of cases [1,12,36,37]. Studies have previously established that there is a strong linear relationship between BP and BMI in growing children. A retrospective cohort study involving 101,606 participants aged 3‒17 y from 3 health systems across the United States indicated that transitioning from obesity to either overweight or normal weight classification was linked to BP reduction, suggesting that a potential lowering of weight could reverse the pathologic increase in BP [35]. However, BMI is known to be strongly correlated with lean mass [11,35]. An earlier study that examined the association between BP and body compositions, measured with dual-energy X-ray absorptiometry, identified lean mass as the strongest predictor of SBP, independent of fat mass [6,8]. This partly explains the lack of a significant linear association between SBP and WHtR categories in under-25-y-olds in this study, as WHtR has the weakest correlation with lean mass in this age group. A similar absence of risk in this age group was also explained in a recent study in the United Kingdom [11,38].

Nonetheless, we observed that the odds of elevated BP were 1.6 times higher among individuals in the high-fat group and twice as high among those in the excess fat category, highlighting the independent effect of excess adiposity on the development of hypertension in the young. In a previous study conducted among first-grade, 7-y-old Taiwanese children, a relatively stronger association with BP was reported (OR: 3.10; 95% CI: 2.05, 4.68) in the fourth quartile with a mean WHtR of 0.54 [39]. Unlike the present study, a significant effect was detected as low as the second quartile (mean WHtR of 0.47), probably due to racial and lifestyle population differences [39]. Previous studies have related nonvalidated WHtR categories with the risk of high BP with varying results [[13], [14], [15], [16], [17]]. In 2 Chinese cohort studies, participants with a mean age of 47.8 y and 51.2 y, WHtR values were categorised into equal tertiles [22,27]. The studies reported that the most significant association with high BP was found with WHtR values >0.53, whereas categorisation of WHtR into quartiles in a Korean cohort (participants aged 40–70) reported an equivalent strength of association at a WHtR cut point of 0.51 [22,23,27]. A recent study from the United Kingdom reported that the WHtR cut point of ≥0.50 was associated with higher SBP in young adults with persistent exposure or adult-onset excess adiposity [38].

A previous study involving 3566 participants from the NHANES 2011–2018 cycle cohort reported that an arbitrary WHtR cutoff of 0.5 classified 85.9%‒97.8% of participants as centrally obese across various ethnic groups [40]. Consequently, the study proposed an optimal WHtR cutoff range between 0.55 and 0.59, with a maximum specificity of 77%, and recommended these cutoffs for predicting cardiometabolic risk factors [40]. In contrast, based on the pediatric WHtR cutpoints applied in this study, 24.6% had normal fat, 9.7% had high fat, and 65.6% had excess fat in the NHANES 2021–2023 cycle of a similar United States cohort. A recent longitudinal study concluded that these new WHtR cutpoints correctly diagnose both total fat mass (specificity: 93% in males and 95% in females) and central (truncal) fat mass, as well as avoiding overdiagnosis of overweight and obesity, in the young population [11,16]. Furthermore, new studies externally validated these new cutoffs, especially the high fat mass category as a predictor of the risk of type 2 diabetes, liver steatosis and fibrosis, and bone fractures in multiracial populations [[16], [17], [18]]. A high WHtR not only signifies body fat above the optimal range but also reflects abnormal fat distribution as well as a contributing pathology to metabolic syndrome risk [30]. The current study further buttresses the recent consensus statements and frameworks that have emphasised the transitioning away from BMI-guided assessment and management of obesity, which must be inexpensively confirmed with WHtR [13,14].

Consistent with the foregoing, we observed that WHtR excess fat was significantly stronger in detecting the risk of hypertension than BMI. This is akin to a recent secondary analysis of a randomised controlled trial, which reported that the BMI-obesity paradox does not exist in the prediction of heart failure when WHtR is used as an index of assessing adiposity [41]. The randomised controlled trial study concluded that patients at risk of heart failure should be identified with WHtR, irrespective of ethnicity, rather than with BMI. Our present study also showed that the WHtR excess fat mass category predicted the risk of elevated BP in non-Hispanic White, non-Hispanic Blacks, and Asians, including several other unspecified minority ethnic groups, but predicted hypertension only in non-Hispanic White. Multiracial evidence from >400,000 UK Biobank adults suggested that guideline-recommended BP thresholds may overestimate elevated BP risks in the Black population and underestimate BP risks among South Asians; however, the WHtR excess fat cutpoint appeared to similarly identify elevated BP risk in these ethnic groups, underscoring the universality of the new pediatric WHtR cutpoint [15,28,41].

The study is limited by its cross-sectional design, since no temporal or causal inferences can be concluded. In addition, future research can investigate the performance of WHtR estimated fat mass categories in novel CVD risk prediction scores. Considering the relatively few participants with hypertension among youth despite doubling the sample size, the null result of the relationship between WHtR and hypertension may be inconclusive, warranting a larger sample size and longitudinal studies.

In conclusion, excess adiposity estimated with WHtR was associated with a higher risk of elevated BP and hypertension. Among children, adolescents, and young adults, WHtR excess adiposity was significantly associated with the risk of elevated BP. Middle-aged adulthood seems critical to mitigating adiposity-induced elevated BP and hypertension risk. WHtR is an inexpensive and universally accessible tool that could replace BMI in screening, prevention, diagnosis, and management of obesity and its CVD sequelae.

## Author contributions

The authors’ responsibilities were as follows– AOA: concept, design, and acquisition of data; DRC: statistical analysis; MWA: initial drafting of the manuscript; MWA, DRC, AOA: interpretation of data, critical revision, and finalization of the manuscript for important intellectual content; AOA: obtained funding. This publication is the work of the authors, and AOA will serve as guarantor for the contents of this paper. All authors had full access to all the data in the study and take responsibility for the integrity of the data and the accuracy of the data analysis; and all authors: read and approved the final manuscript.

## Data availability

The NHANES data is publicly available via this website https://wwwn.cdc.gov/nchs/nhanes/default.aspx.

## Funding

AOA’s research group (UndeRstanding FITness and Cardiometabolic Health In Little Darlings; https://urfit-child.com/) was funded by the Jenny and Antti Wihuri Foundation (grant number: 00180006), the North Savo Regional Finnish Cultural Foundation (grant number: 65191835), the Finnish Cultural Foundation (grant numbers: 00200150, 00230190, and 00250189), Orion Foundation, Aarne Koskelo Foundation, Antti and Tyyne Soininen Foundation, Kuopio University Foundation, Paulo Foundation, Paavo Nurmi Foundation, Yrjö Jahnsson Foundation (grant number: 20217390), Ida Montin Foundation (grant number: 20230289), Fund of Eino Räsänen and Fund of Matti and Vappu Maukonen via the Faculty of Health Sciences University of Eastern Finland, Pediatric Research Foundation (240417), the Finnish Foundation for Cardiovascular Research (grant numbers: 220021, 230012 and 240003), the Alfred Kordelin Foundation (230082) and the Novo Nordisk Foundation (NNF24SA0090437 and NNF25SA0104079 ). The funders had no role in the design and conduct of the study; collection, management, analysis, and interpretation of the data; preparation, review, or approval of the manuscript; and decision to submit the manuscript for publication.

## Conflict of interest

The authors report no conflicts of interest.
