# Supplementary material for: Novel Waist-to-Height Ratio Estimated Fat Mass Pediatric Cut-offs Predict Hypertension Better than Body Mass Index in Multiracial United States Youths and Adults: The National Health and Nutrition Examination Survey 2015–2023 Cycle
Source: J Nutr. 2026 Feb 18;156(4):101426. doi: 10.1016/j.tjnut.2026.101426 (PMC13084610; doi:10.1016/j.tjnut.2026.101426)
Supplement: multimedia component 1 [file mmc1.pdf]

Supplemental Appendix

Novel Waist-to-Height Ratio Estimated Fat Mass Pediatric Cut-offs Predict Hypertension Better than Body Mass Index in Multiracial US Youths and Adults: The NHANES 2017 – 2023 Cycle

Brief title: Waist-to-height ratio with hypertension

Mahidere W. Ali[1], Douglas R. Corsi[2], Andrew O. Agbaje[1,3]

[1]Institute of Public Health and Clinical Nutrition, School of Medicine, Faculty of Health Sciences, University of Eastern Finland, Kuopio, Finland

[2]Rutgers Robert Wood Johnson Medical School, Department of Medicine, New Brunswick New Jersey, US

[3]Children's Health and Exercise Research Centre, Department of Public Health and Sports Sciences, Faculty of Health and Life Sciences, University of Exeter, Exeter, UK.

Address correspondence to:
Andrew O. Agbaje, MD, MPH, PhD, FACC, FESC, FAHA, FNYAM, Cert. Clinical Research *(Harvard)*
Professor (associate) of Clinical Epidemiology and Child Health
Institute of Public Health and Clinical Nutrition, School of Medicine, Faculty of Health Sciences, University of Eastern Finland, Kuopio Campus.
Address: Yliopistonranta 8, P.O. Box 1627, 70211 Kuopio, Finland
E-mail: andrew.agbaje@uef.fi
Website: https://urfit-child.com/

Supplemental Table 1 Characteristics of the NHANES Cohort 2021-2023 Survey cycle

	**Whole Cohort**	**<25-year-olds**
Characteristic	Mean (SD)	Mean (SD)
Participants (no.)	7243	1886
Age (years)	44.8 (22.6)	14.7 ± 4.5
Sex (Female) n (%)	3881 (56.6)	946 (50.2)
Height (cm)	164.8 (12.1)	158.9 ± 15.1
Weight (kg)	77.5 (24.2)	61.4 ± 23.6
Waist circumference (cm)	96.0 (19.1)	80.2 ± 17.0
BMI (kg/m²)	28.2 (7.5)	23.7 ± 6.8
Waist-to-height ratio	0.58 (0.1)	0.51 ± 0.1
Heart rate (bpm)	72.2 ± 12.6	76.5 ± 21.9
Systolic blood pressure (mmHg)	119.0 (18.0)	106.4 ± 10.6
Diastolic blood pressure (mmHg)	72.2 (11.4)	64.2 ± 8.6
Total cholesterol (mg/dL)	192.4 (45.1)	158.4 ± 30.75
hs-CRP (mg/L)	3.7 (6.7)	2.5 ± 5.2
Smoking Status n (%) (Do you now smoke cigarettes)	2462 (34.0)	672 (35.6)
Education n (%)		
Less than 9th grade	263 (4.7)	635 (33.7)
9–11th grade	433 (7.7)	40 (21.2)
High school/GED	1,186 (21.0)	289 (15.3)
Some college/AA	1,720 (30.5)	326 (17.3)
College grad or above	2,038 (36.1)	569 (30.2)
Race/Ethnicity n (%)		
Mexican American	619 (8.5)	277 (10.9)
Other Hispanic	806 (11.1)	275 (10.8)
Non-Hispanic White	4,001 (55.2)	775 (30.6)
Non-Hispanic Black	894 (12.3)	262 (10.3)
Non-Hispanic Asian	418 (5.8)	129 (5.1)
Other/Multi-Racial	505 (7.0)	168 (6.6)
Sedentary time (min/day)	416.1 (737.3)	311.0\ (161)

WHtR, waist-to-height ratio; BP, blood pressure; GED, General Educational Development; AA, Associate of Arts; hs-CRP, high-sensitivity C-reactive protein. A two-sided p-value <0.05 was considered statistically significant. Values are presented as mean (standard deviation, SD) for continuous variables and n (%) for categorical variables.

Supplemental Table 2 Characteristics of the children, adolescents and young adults (<25 years old) from the NHANES Cohort 2021-2023 Survey cycle

**Characteristic**	**WHtR (0.40 – <0.50) Normal fat (n= 1095, 58.1%)**	**WHtR (0.50 – <0.53) High fat (n= 184, 9.8%)**	**WHtR (>0.53) Excess fat (n= 607, 32.2%)**	**p-value**
Age (years)	14.2 ± 4.3	15.3 ± 4.6	15.3 ± 4.5	<0.001
Sex (Female)	521 (47.6)	108 (58.7)	317 (52.2)	<0.001
Height (cm)	158.3 ± 16.1	159.7 ± 13.9	159.9 ± 13.5	0.087
Weight (kg)	50.5 ± 15.7	62.6 ± 15.5	80.8 ± 24.8	<0.001
Waist circumference (cm)	69.7 ± 8.5	82.0 ± 7.3	98.6 ± 14.6	<0.001
BMI (kg/m²)	19.6 ± 3.1	24.0 ± 2.5	30.9 ± 6.3	<0.001
Waist-to-height ratio	0.44 ± 0.03	0.51 ± 0.01	0.62 ± 0.07	<0.001
Heart rate (bpm)				
Systolic blood pressure (mmHg)	105.8 ± 10.2	107.5 ± 11.4	107.1 ± 10.9	0.019
Diastolic blood pressure (mmHg)	62.2 ± 7.8	65.1 ± 8.4	67.4 ± 9.0	<0.001
Elevated blood pressure	222 (20.3%)	58 (31.5%)	225 (37.1%)	<0.001
Hypertension	5 (0.5%)	3 (1.6%)	10 (1.6%)	
Total cholesterol (mg/dL)	155.8 ± 29.4	161.1 ± 33.3	162.5 ± 31.8	<0.001
hs-CRP (mg/L)	1.1 ± 2.8	2.4 ± 4.8	4.9 ± 7.3	<0.001
Smoking Status (Do you now smoke cigarettes)	6 (0.5)	3 (1.6)	8 (1.3)	<0.001
Education				
Less than 9th grade	418 (38.2)	59 (32.1)	158 (26.0)	
9–11th grade	13 (1.2)	2 (1.1)	25 (4.1)	
High school/GED	150 (13.7)	32 (17.4)	107 (17.6)	
Some college/AA	168 (15.3)	34 (18.5)	124 (20.4)	
College grad or above	346 (31.6)	57 (31.0)	193 (31.8)	
Race/Ethnicity				<0.001
Mexican American	130 (11.9)	35 (3.2)	112 (10.5)	
Other Hispanic	140 (12.8)	26 (2.4)	109 (10.2)	
Non-Hispanic White	483 (44.1)	72 (6.6)	220 (20.7)	
Non-Hispanic Black	153 (14.0)	19 (1.7)	90 (8.5)	
Non-Hispanic Asian	90 (8.2)	19 (1.7)	20 (1.9)	
Other/Multi-Racial	99 (9.0)	13 (1.2)	56 (5.2)	
Sedentary time (min/day)	411 (138)	410 (132)	403 (152)	0.52

WHtR, waist-to-height ratio; BMI, body mass index; GED, General Educational Development; AA, Associate of Arts; hs-CRP, high-sensitivity C-reactive protein. Values are presented as mean ± standard deviation (SD) for continuous variables and n (%) for categorical variables. Comparisons across WHtR categories were performed using one-way analysis of variance (ANOVA) for continuous variables and chi-square (χ^2) tests for categorical variables. A two-sided p-value <0.05 was considered statistically significant.

Supplemental Table 3 Associations of waist-to-height ratio estimated fat mass cutpoints with elevated blood pressure and hypertension in <25-year-olds.

**Adiposity Measure**	**Elevated BP (≥120/70 mmHg)**		**Hypertension (≥140/90 mmHg)**	
Frequency (%)	505 (27.0)		18 (1.0)	
Model 1	***Odds ratio (95% CI)***	p-value	***Odds ratio (95% CI)***	p-value
WHtR (0.40–<0.50) Normal fat	*Reference*	-	*Reference*	-
WHtR (0.50–<0.53) High fat	1.81 (1.28 – 2.56)	<0.001	3.61 (0.86 – 15.23)	0.081
WHtR (≥0.53) Excess fat	2.32 (1.86 – 2.89)	<0.001	3.65 (1.24 – 10.73)	0.019
Model 2				
WHtR (0.40–<0.50) Normal fat	*Reference*	-	*Reference*	-
WHtR (0.50–<0.53) High fat	1.70 (1.11 – 2.61)	0.015	2.58 (0.54 – 12.31)	0.234
WHtR (≥0.53) Excess fat	2.16 (1.59 – 2.92)	<0.001	2.33 (0.73 – 7.41)	0.154

WHtR, waist-to-height ratio; BP, blood pressure; CI, confidence interval. Odds ratios (ORs) and 95% confidence intervals (CIs) were calculated using logistic regression models. Model 1 is unadjusted. Model 2 is adjusted for age, sex, heart rate, educational status, smoking status, race, sedentary time, moderate physical activity, fasting total cholesterol, and high-sensitivity C-reactive protein. A two-sided p-value <0.05 was considered statistically significant.

Supplemental Table 4 Comparing Waist-to-Height Ratio with Body Mass Index for Predicting Hypertension

Adiposity Measure	Elevated Blood Pressure (≥120/70 mmHg)		Hypertension (≥140/90 mmHg)	
	Odds Ratio (95% CI)	P-value	Odds Ratio (95% CI)	P-value
**Waist-to-Height Ratio Categories**				
Normal fat mass (0.40–<0.50)	*Reference*	—	*Reference*	—
High fat mass (0.50–<0.53)	1.49 (1.04 – 2.12)	**0.001**	1.82 (1.49 – 2.22)	**<0.001**
Excess fat mass (≥0.53)	1.91 (1.40 – 2.59)	**<0.001**	2.61 (2.21 – 3.08)	**<0.001**
**Body Mass Index Categories**				
Normal weight	*Reference*	—	*Reference*	—
Overweight	1.73 (1.50 – 2.00)	**<0.001**	1.11 (0.90 – 1.36)	0.321
Obesity	2.48 (2.11 – 2.91)	**<0.001**	1.25 (1.00 – 1.56)	0.058

WHtR, waist-to-height ratio; BMI, body mass index; CI, confidence interval; BP, blood pressure. Odds ratios (ORs) and 95% confidence intervals (CIs) were calculated using logistic regression models. All models were adjusted for age, sex, heart rate, educational status, smoking status, race, sedentary time, moderate physical activity, fasting total cholesterol, and high-sensitivity C-reactive protein. A two-sided p-value <0.05 was considered statistically significant.

Supplemental Table 5 Characteristics of NHANES 2017–2018 and 2021–2023 participants aged 12–24 years, by waist-to-height ratio adiposity categories

Characteristic	Normal fat (n = 1,418)	High fat mass (n = 390)	Excess fat mass (n = 1,011)	p-value
Age, years, mean (SD)	16.48 (3.33)	16.98 (3.45)	17.54 (3.66)	<0.001
Systolic BP, mmHg, mean (SD)	108.92 (10.30)	109.76 (10.48)	109.07 (10.79)	0.396
Diastolic BP, mmHg, mean (SD)	63.60 (7.83)	66.34 (7.58)	68.52 (8.72)	<0.001
Elevated BP, n (%)	339 (23.9%)	132 (33.8%)	408 (40.4%)	
Hypertension, n (%)	12 (0.8%)	5 (1.3%)	14 (1.4%)	
Waist circumference, cm, mean (SD)	72.73 (6.37)	83.61 (5.60)	102.84 (13.57)	<0.001
Height, cm, mean (SD)	166.88 (10.28)	164.48 (10.11)	165.38 (9.50)	<0.001
BMI, kg/m², mean (SD)	20.43 (2.60)	24.49 (2.13)	32.37 (6.19)	<0.001
**Sex, n (%)**				<0.001
Male	795 (56.1)	154 (39.5)	467 (46.2)	
Female	623 (43.9)	236 (60.5)	544 (53.8)	
**Race/ethnicity, n (%)**				<0.001
Mexican American	181 (12.8)	75 (19.2)	228 (22.6)	
Other Hispanic	152 (10.7)	46 (11.8)	133 (13.2)	
Non-Hispanic White	540 (38.1)	145 (37.2)	320 (31.7)	
Non-Hispanic Black	274 (19.3)	51 (13.1)	181 (17.9)	
Other race/multiracial	271 (19.1)	73 (18.7)	149 (14.7)	
**Parental education, n (%)*†**				0.523
< 9th grade	3 (1.2)	1 (1.1)	4 (1.3)	
9–11th grade	24 (9.6)	8 (9.0)	35 (11.3)	
High school/GED	73 (29.1)	28 (31.5)	114 (36.8)	
Some college/AA degree	103 (41.0)	34 (38.2)	115 (37.1)	
College graduate+	48 (19.1)	18 (20.2)	42 (13.5)	
Income-to-poverty ratio, mean (SD)	2.38 (1.62)	2.20 (1.63)	1.96 (1.39)	<0.001
Total cholesterol, mg/dL, mean (SD)	152.58 (28.23)	160.19 (31.21)	167.42 (33.66)	<0.001
Glucose, mg/dL, mean (SD)	95.61 (14.28)	95.95 (7.34)	98.71 (13.71)	0.001
hs-CRP, mg/L, mean (SD)	1.15 (3.30)	2.04 (5.94)	4.13 (7.99)	<0.001
Sedentary time, min/day, mean (SD)	400.55 (641.52)	401.24 (773.55)	391.82 (641.88)	0.975
Physical activity (harmonized), mean (SD)	52.29 (94.17)	61.13 (98.77)	76.06 (111.58)	<0.001

* Education among parents/household adults of youth participants, as defined in NHANES.
† Percentages may not sum to 100 due to rounding.

Supplemental Table 6: Characteristics of NHANES 2017–2018 participants aged 12–24 years, by waist-to-height ratio adiposity categories

Characteristic	Normal fat (n = 710)	High fat mass (n = 190)	Excess fat mass (n = 544)	p-value
Age, years, mean (SD)	16.58 (3.39)	16.80 (3.20)	17.76 (3.73)	<0.001
Systolic BP, mmHg, mean (SD)	109.74 (10.44)	110.42 (9.70)	109.46 (10.69)	0.582
Diastolic BP, mmHg, mean (SD)	63.88 (7.86)	66.31 (6.90)	68.26 (8.39)	<0.001
Elevated BP, n (%)	171 (24.1%)	58 (30.5%)	216 (39.7%)	<0.001
Hypertension, n (%)	8 (1.1%)	1 (0.5%)	5 (0.9%)	0.43
Waist circumference, cm, mean (SD)	72.53 (6.27)	83.82 (5.54)	103.04 (13.54)	<0.001
Height, cm, mean (SD)	166.49 (10.28)	164.74 (10.08)	165.36 (9.46)	0.037
BMI, kg/m², mean (SD)	20.39 (2.54)	24.47 (2.18)	32.35 (6.17)	<0.001
**Sex, n (%)**				<0.001
Male	410 (57.7)	76 (40.0)	251 (46.1)	
Female	300 (42.3)	114 (60.0)	293 (53.9)	
**Race/ethnicity, n (%)**				<0.001
Mexican American	97 (13.7)	41 (21.6)	133 (24.4)	
Other Hispanic	58 (8.2)	16 (8.4)	51 (9.4)	
Non-Hispanic White	227 (32.0)	63 (33.2)	153 (28.1)	
Non-Hispanic Black	179 (25.2)	28 (14.7)	115 (21.1)	
Other race/multiracial	149 (21.0)	42 (22.1)	92 (16.9)	
**Parental education, n (%)*†**				0.684
< 9th grade	2 (1.5)	1 (2.7)	4 (2.2)	
9–11th grade	21 (15.4)	5 (13.5)	19 (10.3)	
High school/GED	40 (29.4)	14 (37.8)	71 (38.6)	
Some college/AA degree	56 (41.2)	15 (40.5)	68 (37.0)	
College graduate+	17 (12.5)	2 (5.4)	22 (12.0)	
Income-to-poverty ratio, mean (SD)	2.26 (1.57)	2.11 (1.60)	1.94 (1.39)	0.003
Total cholesterol, mg/dL, mean (SD)	154.01 (27.40)	156.67 (28.46)	167.98 (34.47)	<0.001
Glucose, mg/dL, mean (SD)	96.54 (8.72)	97.27 (6.39)	100.36 (16.83)	0.001
hs-CRP, mg/L, mean (SD)	1.30 (3.53)	2.17 (7.26)	4.20 (6.80)	<0.001
Sedentary time, min/day, mean (SD)	371.04 (635.07)	325.67 (174.67)	367.96 (607.09)	0.83
Physical activity (harmonized), mean (SD)	43.22 (104.64)	53.55 (104.47)	75.83 (127.76)	<0.001

* Education among parents/household adults of youth participants, as defined in NHANES.
† Percentages may not sum to 100 due to rounding.
